# Rapid Assessment of Oxidative Damage Potential: A Comparative Study of Engineered Stone Dusts Using a Deoxyguanosine Assay

**DOI:** 10.3390/ijerph19106221

**Published:** 2022-05-20

**Authors:** Leigh Thredgold, Chandnee Ramkissoon, Chellan Kumarasamy, Richard Gun, Shelley Rowett, Sharyn Gaskin

**Affiliations:** 1 Adelaide Exposure Science and Health, School of Public Health, University of Adelaide, Adelaide, SA 5005, Australia; leigh.thredgold@adelaide.edu.au (L.T.); chandnee.ramkissoon@adelaide.edu.au (C.R.); chellan.kumarasamy@curtin.edu.au (C.K.); richard.gun@adelaide.edu.au (R.G.); 2 SafeWork SA, Government of South Australia, Adelaide, SA 5035, Australia; msrowett@bigpond.com.au

**Keywords:** engineered stone, artificial stone, reactivity, oxidative damage, deoxyguanosine, respirable crystalline silica

## Abstract

The popularity of engineered stone (ES) has been associated with a global increase in occupational lung disease in workers exposed to respirable dust during the fabrication of benchtops and other ES products. In this study, the reactivity and subsequent oxidative reduction potential of freshly generated ES dusts were evaluated by (i) comparing different engineered and natural stones, (ii) comparing settled and respirable stone dust fractions and (iii) assessing the effect of ageing on the reactivity of freshly generated stone dust. An established cell-free deoxyguanosine hydroxylation assay was used to assess the potential for oxidative DNA damage. ES dust exhibited a higher relative reactivity than two of the three natural stones tested. Respirable dust fractions were found to be significantly more reactive than their corresponding settled fraction (ANOVA, *p* < 0.05) across all stone types and samples. However, settled dust still displayed high relative reactivity. The lower reactivity of the settled dust was not due to decay in reactivity of the respirable dust when it settled but rather a result of the admixture of larger nonrespirable particles. No significant change in respirable dust reactivity was observed for three ES samples over a 21-day time period, whereas a significant decrease in reactivity was observed in the natural stone studied. This study has practical implications for dust control and housekeeping in industry, risk assessment and hazard management.

## 1. Introduction

Over the past two decades, engineered stone (ES), also known as artificial or composite stone, has become an increasingly popular, more affordable alternative to natural stone products (i.e., granite and marble) for benchtops in domestic and commercial settings. It is predicted that the global value of the engineered stone industry will increase from USD 21.5 billion to 30.6 billion from 2020 to 2027 [1]. This rapid expansion has led to a substantial increase in the number of workers employed in this industry and the re-emergence of a number of respiratory issues amongst this workforce, including occupational lung disease, silicosis, as well as variations of it [2,3]. This is currently a global issue with major loci in Australia, Israel, Spain and USA, with cases also observed in multiple other countries [4,5]. Since the introduction of ES in 1986, an increasing number of work-related silicosis cases have been reported in these countries [4,6]. Silicosis is a chronic fibrotic disease (pneumoconiosis), traditionally associated with long latency periods correlating with long-term exposure to respirable crystalline silica (RCS) and typically presents in older workers. Within the engineered stone industry, evidence has emerged of a form of ‘accelerated silicosis’, which has shown shorter latency periods in highly exposed workers [7,8,9,10,11]. The popularity of these products and the upwards trajectory of the industry resulting in higher rates of occupational disease necessitates that the toxicity of dust generated during ES fabrication be better understood.

A major barrier in understanding the toxicity of engineered stone dusts is the inherent variability of these manufactured materials, as their composition is necessarily varied to create various patterns, colours and designs to align with consumer trends. This leads to a diverse range of ES products that can vary in pigment, resin and mineral composition not only between manufacturers but within the same manufacturer. All of these products have high crystalline silica contents (typically >90% by weight). Few studies have assessed the genotoxicity of natural mineral dusts, and no such investigation assessing the genotoxicity potential of ES dusts has been conducted [12]. As a better-characterised hazard, natural stone dusts offer a baseline against which ES dusts can be compared. In addition, the potential difference in genotoxicity between airborne respirable ES dust generated during fabrication processes and that of settled dusts deposited on workplace surfaces and floors, which may be resuspended at a later time, is yet to be explored. The association between exposure to particulates (PM10) and lung cancer has been demonstrated in epidemiological studies of urban populations [13]. A possible mechanism is the generation of adducts, which may lead to cumulative mutations and eventually to the generation of cancer cells.

In vitro studies have suggested that RCS induces genotoxicity via intracellular production of reactive oxygen species (ROS) [14,15,16]; however, the exact genotoxic modes of action in vivo are not well understood and still under investigation [17]. Given the high crystalline silica content of engineered stone products (>90% by weight), assessing the reactivity of ES dusts combined with their ability to produce ROS and cause oxidative damage to DNA is one way to evaluate the genotoxic potential of these materials to cause damage in the body. The deoxyguanosine (dG) assay is a simple, well-accepted in vitro method that uses ROS-mediated hydroxylation of 2′-deoxyguanosine to 8-hydroxy-2′-deoxyguanosine as an indicator of oxidative DNA damage [18,19,20,21]. The conversion simulates, in vitro, the hydroxylation of the guanine base of DNA in the body, representing oxidative lesion formation, which, in turn, is indicative of DNA damage or mutagenesis [22]. Therefore, 8-hydroxy-2′-deoxyguanosine is a well-recognised standard marker for oxidative DNA damage [23,24,25,26,27,28]. The dG assay has previously been applied to assess the reactivity of mineral fibres (i.e., asbestos), particulate matter and air pollutants such as fly ash, in the presence of hydrogen peroxide, as an indicator of their genotoxic potential [20,21,26,27,28]. These studies have demonstrated the reactive nature of these materials to produce ROS and cause oxidative damage as an indicator of genotoxicity, suggesting that the dG assay has the potential to be a rapid screening tool to assess the reactivity of ES dusts.

This study aimed to address current knowledge gaps around the reactivity of freshly generated ES dusts by applying them to a cell-free in vitro deoxyguanosine assay. The data generated gives insight into the ability of these materials to produce ROS and cause subsequent oxidative damage to a molecule found within DNA. Applying the same assay to freshly generated natural stone dusts allows for a comparative assessment of oxidative damage potential across a range of naturally occurring and manufactured materials. Further, this study aimed to assess whether there is any difference in reactivity between airborne respirable dust and dust that has “settled” onto workplace surfaces, along with the influence of ageing on the reactivity of stone dusts. This study demonstrates the applicability of a standardised deoxyguanosine assay as a screening tool for assessing the comparative reactivity of ES dusts. Outcomes may help inform (i) the pathological risk posed by these dusts with regards to oxidative damage potential and (ii) further research to better understand the toxicity presented by these materials.

## 2. Materials and Methods

### 2.1. Stone Samples

A total of 12 engineered stone samples (ES1–ES13) were chosen from amongst the product lines and brands holding the highest volume of sales in Australia and were procured from commercial suppliers. Three natural stone samples (NS1–NS3) were procured from local stonemasons and assessed alongside the engineered stone samples. Two additional materials were assessed alongside the stones in this study for comparative purposes, namely, a respirable crystalline silica reference material and the common concrete building material Hebel. All samples are outlined in Table 1, along with previously published physicochemical properties [29] of respirable dust generated from each material.

### 2.2. Reagents

Sodium citrate, citric acid, acetic acid, hydrogen peroxide, sodium hydroxide and methanol were sourced from VWR Chemicals (Tingalpa, Australia), and 2′-Deoxyguanosine, 8-hydroxy-2′-deoxyguanosine (8-OH-dG) and phosphate buffered saline (PBS) were sourced from Sigma-Aldrich (Sydney, Australia). Respirable crystalline silica (NIST^®^ Standard Reference Material 1878b; 96.73% alpha quartz) was procured from Sigma-Aldrich (Sydney, Australia). Ultrapure water (IBIS Plus 6 Iph, IBIS Technology, Perth, Australia) was used for the preparation of all reagents.

### 2.3. Generation of Stone Dust

Generation of ES dust was achieved using simulated workplace conditions to mimic dust generated in fabrication workshops. An industry-appropriate angle grinder (Metabo; W72100, 720 watts) fitted with a Diarex ultra continuous diamond blade was used to perform continuous zip cuts on each stone sample to generate sufficient stone dust for analysis. The simulated stone cutting process was isolated in a custom-made Perspex chamber to prevent exposure of the operator to respirable dust. Sampling of respirable dust was carried out using SKC Parallel Particulate Impactor (PPI) dust sampling heads, and Casella Higgins-Dewell Cyclone (11600B) dust sampling heads, in tandem, placed inside the chamber, connected to an SKC Leland Legacy pump (flow rate: 8 L/min) and an SKC AirCheck TOUCH pump (flow rate: 2.2 L/min), respectively. Dust sampling was conducted for 15 min per sample, with samples of settled dust collected from surfaces inside the chamber after each cutting event.

### 2.4. Assessment of Dust Oxidative Damage Potential

**Figure 1:**
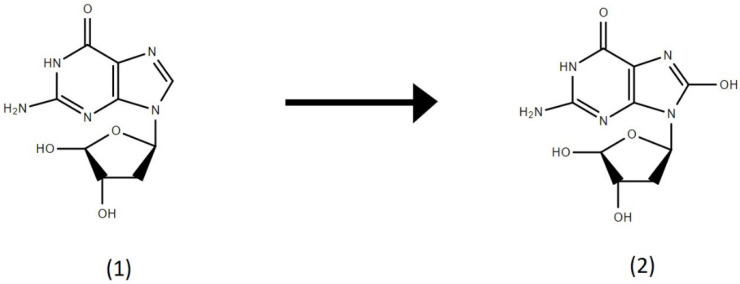
Conversion of (**1**) 2′-deoxyguanosine to (**2**) 8-hydroxy-2′-deoxyguanosine.

The potential for actively generated stone dusts to generate ROS and cause subsequent oxidative damage was assessed using a cell-free deoxyguanosine assay. The protocol for the deoxyguanosine assay was adapted from previously published methods assessing the reactivity of particulate matter [27,28]. The ROS-mediated hydroxylation reaction of this assay is shown in Figure 1.

Specifically, five milligrams of freshly generated dust were suspended in 3.8 mL of 0.1 M PBS (pH 7.2), to which 1 mL of 0.2 M H_2_O_2_ and 200 µL of 0.01 M 2′-deoxyguanosine were added. Assay samples were incubated on an orbital shaker in the dark with caps removed to facilitate air exchange for 60 min. After incubation, all samples were filtered through a 0.45 µm filter prior to analysis via HPLC-EC. All samples were analysed in duplicate alongside a negative control (containing no stone dust). Samples were analysed in duplicate due to limitations in the collection of higher quantities of respirable dust.

### 2.5. Ageing of Stone Dusts

Dusts in commercial workplaces often settle on surfaces and floors and can remain in place for extended time periods if housekeeping practices are poor. To determine the effect of age on the oxidative damage potential of stone dust, five engineered stone samples with varying crystalline silica content were chosen for analysis. Each dust sample was actively generated as described above (s2.3), and the settled fraction was collected, divided into 5 mg subsamples and stored with air exchange for up to 21 days. The oxidative damage potential of aged samples was determined by subjecting duplicate samples of each dust to the deoxyguanosine assay on days 0, 1, 7, 14 and 21 to observe if any decay in reactivity resulted from the ageing process.

### 2.6. Determination of 8-Hydroxy-2′-deoxyguanosine

Analysis of 8-hydroxy-2′deoxyguanosine was performed using high-performance liquid chromatography with electrochemical detection (HPLC-EC), namely a Perkin Elmer Flexar^TM^ LC system (Perkin Elmer, Melbourne, Australia) coupled to a Waters 2465 Electro-Chemical Detector (Waters, Sydney, Australia). The system employed a Phenomonex Kinetex^®^ C18 column (150 × 4.6 mm 5 µm) and was run using a sodium acetate buffer (sodium acetate, 25 mM; acetic acid, 10 mM; citric acid, 12.5 mM; sodium hydroxide, 30 mM) with 0.1% *v*/*v* methanol at a flow rate of 1 mL/min. Electrochemical (EC) detection was carried out at an electrode oxidation potential (EC) of +0.6 V, with a range of 100 nA and a 5% offset.

### 2.7. Data Analysis

Results of the assay were expressed as µg of 8-OH-dG generated per 10^5^ dG for each stone sample. Differences between aged samples were analysed via one-way analysis of variance (ANOVA) with Duncan post hoc test. The unpaired *t*-test was used to assess differences between the respirable and settled fraction of identical stone samples. All differences with a *p*-value of <0.05 were considered statistically significant. All statistical analysis was carried out using SPSS v27.0.0.0 (IBM, Armonk, NY, USA).

## 3. Results

### 3.1. Physicochemical Properties of Stone Dusts

The physicochemical properties of the stone dust samples used in this study, with the exception of ES12 and Hebel, have been reported previously [29] and are displayed in Table 1 for reference. In summary, all respirable engineered stone dust contained high silica content (as quartz or cristobalite), ranging from 47.2% to 91%, compared with respirable natural stone dust, which ranged from 3.50% to 30.1%. The traditional building material Hebel was found to contain 5.9% silica. Engineered stone dusts contained between 8.62% and 20.0% resin. The particle size of all respirable dust samples was comparable and ranged between approximately 200 and 715 nm in diameter. Further, the reported zeta potential of each stone dust ranged between −15.2 and −33.8 mV, with the majority of dusts >−25 mV. All stone dusts contained trace and minor elements, with aluminium the most abundant, apart from silicon, the exception being NS1, which contained high amounts of iron (6.63%) and aluminium (8.36%) when compared with all other stone dusts. The complementary physicochemical properties of dusts generated from two additional materials were determined in this study, namely ES13 and Hebel. The data for these dusts are presented alongside the previously reported data in Table 1 for comparative purposes.

### 3.2. Oxidative Damage Potential of Freshly Generated Stone Dusts

The potential for stone samples, as freshly generated respirable and settled dust, to cause oxidative damage was measured using a deoxyguanosine assay. This was measured as the ability of generated dusts to facilitate the formation of hydroxyl radicals leading to the conversion of 2′-deoxyguanosine to 8-hydroxy-2′-deoxyguanosine, with the results shown in Table 2. The highest recorded reactivity was for the respirable fraction of ES7 (4757 ± 853), whereas the lowest was achieved for the purchased RCS NIST standard (14 ± 25). A degree of variability was observed in the reactivity of the respirable engineered stone dust samples, although all respirable dust from engineered stone gave comparatively high values in the assay, indicating their potential to cause oxidative damage. Two of the three natural stone samples (NS2 and NS3) gave low reactivity values for respirable and settled dust when compared with those of engineered stones, whilst the third (NS1) was on par with the measured reactivity of the engineered stone samples. The building material used in this study (Hebel) also gave a comparatively low reactivity for both respirable and settled dusts. Significantly, a large difference in oxidative damage potential was observed between respirable and settled dust fractions for all materials tested (*p* ≤ 0.01), with this trend being particularly noticeable in engineered stone samples. The one exception was Hebel, in which the low reactivity meant respirable and settled dust fractions were not statistically different (*p* > 0.05).

### 3.3. Effect of Stone Dust Ageing

**Figure 2:**
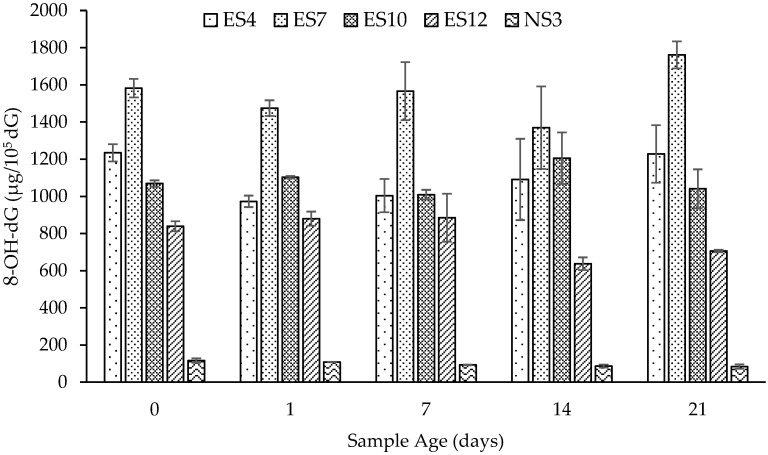
Effect of ageing on the oxidative damage potential of actively generated engineered (ES4, 7, 10 and 12) and natural (NS3) stone dusts measured using the deoxyguanosine assay. Presented as mean ± SD (*n* = 2).

Freshly generated stone dust (settled fraction) was collected and stored open to the atmosphere for up to 21 days. At designated time periods, samples were taken and applied to the deoxyguanosine assay, with the results presented in Figure 2. ES7 exhibited an 11% increase in reactivity, while ES12 exhibited a 15% loss in reactivity. Despite these observations, none of the ES dusts studied exhibited a significant gain or loss in reactivity or oxidative damage potential across the 21-day ageing process (*p* > 0.05). In comparison, the natural stone dust (NS3) did exhibit a statistically significant 28% drop in reactivity across the ageing time frame, from 117 ± 12 at day 0 to 84 ± 9 at day 21 (*p* < 0.05).

## 4. Discussion

This exploratory study evaluated the relative reactivity, via a deoxyguanosine assay, of actively generated engineered stone dusts, both airborne respirable and settled, and compared them with natural stone dusts. The reactivity of ES dusts is of high importance as an indicator of oxidative damage potential (e.g., DNA damage) of these materials. This research has highlighted that airborne respirable ES dusts generated during the cutting of stone using a handheld power tool were up to three times more reactive than dust that settled on surfaces. Further, all ES dusts displayed a higher relative reactivity than two out of the three natural stone dusts tested and were up to 15 times more reactive than dust produced from the common building material Hebel. The effect of ageing on settled dust reactivity was found to be minimal, with no statistical difference over a 21-day period for four ES samples. Whilst the natural stone dust tested (NS3) showed a statistically significant reduction in reactivity over time (*p* < 0.05), this was observed from a considerably lower starting reactivity.

A range of physicochemical properties of the stone dusts used in this study have been previously characterised and are summarised in Table 1 [29]. All samples of ES had a high crystalline silica content (66.0–90.9%), and the corresponding reactivity of the respirable fraction of each dust ranged from 2103 ± 156 to 4757 ± 853 µg/10^5^ dG. In contrast, NS3, with a crystalline silica content of 11.0%, had a corresponding reactivity of 948 ± 141 µg/10^5^ dG, and NS2, with an even lower content of 3.5%, a reactivity of only 162 ± 4.8 µg/10^5^ dG. This is indicative of a strong association between crystalline silica concentration and oxidative damage potential. This hypothesis is further supported by the reactivity of respirable dust produced from the common building material Hebel, which exhibited a much lower reactivity than all ES dusts (312 ± 87 µg/10^5^ dG) and a crystalline silica content of 5.9%. The relatively high reactivity of NS1 (3305 ± 86 µg/10^5^ dG) is anomalous; this sample has an intermediate level of crystalline silica (30.1%), suggesting that the association between silica content and reactivity may be nonlinear. An alternate explanation is that the relatively high reactivity of NS1 is related to its iron content, which is considerably greater than all other samples. The presence of iron and other transition metal ions has been shown to increase the reactivity of airborne pollutants using the deoxyguanosine assay via mediation of hydroxyl radical formation, an effect that is attenuated by the presence of metal ion chelators [26,27].

The low reactivity of the respirable alpha quartz reference standard, initially included as a positive control, was unexpected. However, it is reported that this material is treated with hydrofluoric acid (HF) and then hydrochloric acid during production [30], a process that would result in the ‘stripping’ of metal ions from the crystalline surface. These acid treatments almost certainly reduce any surface reactivity likely to lead to oxidative action. Further, this material was of unknown age, and significant ageing over prolonged time periods may have resulted in a reduction in reactivity [31].

The reactivity of all ES and NS settled dusts was significantly lower (*p* ≤ 0.01) than that of airborne respirable dusts. This reduced reactivity of settled dust is likely a result of the admixture of larger particles not present in the samples of respirable dust. (Note that in order to mitigate the influence of an immediate fall in reactivity, all dusts were applied to the assay within 1 hour of generation for all stone samples.) The settled fraction in this study was dust collected from surfaces postcutting and represented both settled respirable dusts as well as larger size fractions produced from the cutting process. Small particle size has been associated with increased reactivity in previous studies evaluating the reactivity of particulate matter, including environmental particulates (PM10, PM25), diesel, gasoline, woodsmoke soot, coal fly ash and oil fly ash using the deoxyguanosine assay [26,27]. This is an important finding, as primary exposure in fabrication workplaces is to airborne respirable dust, highlighting the importance of controlling dust generated during ES processing at the source of emission.

It is likely that the greater reactivity of smaller particles is a function of a greater specific surface area. Increased surface area, associated with small particle size, tends to be more toxic and cause more stress to alveolar macrophages than bigger particles [32]. Specific surface area may also be a factor in the greater reactivity of ES compared with NS (with the exception of NS3). The mean particle size of respirable ES dust in this study ranged between 218–715 nm. The particle size of NS dusts was comparable (503–634 nm). Notwithstanding the similarity in particle size, previous studies have shown that ES has a higher specific surface area due to the irregular shape of its crystals [29].

All ES dusts exhibited a particle charge, measured as mean zeta potential, of <−25 mV. Of the natural stones, NS1 exhibited a charge of −28.3 mV, whereas NS2 and NS3 were considerably lower, −15.2 and −22.9 mV, respectively, and also had much lower reactivity in the deoxyguanosine assay. Hebel also demonstrated a much lower particle charge (−21.7 mV) and had a considerably lower reactivity, suggesting that particle charge may play a role in determining particle reactivity. However, the mechanisms that govern particle surface charge are complex and often influenced by the surface functionality of crystalline silica, which has been the subject of other studies [33,34,35].

All ES dusts in this study contained 9–20% resin by weight; however, no correlation between resin content and dust reactivity was observed. Previous research has suggested that the reactive pathways of ES dusts may be influenced by the presence of polymeric binding resin [12,36]. Pavan et al. hypothesised that the reactive pathways of ES dust are altered due to resin coating RCS particles after observing a significantly higher capacity for cellular damage in ES dusts that had been stripped of resin via thermolysis [12]. This phenomenon was not explored in this study, as the focus was on measuring the reactivity of actively generated dust. We found ES dust to be more reactive than NS, despite the presence of resin. Furthermore, the presence of resin did not result in any decay in reactivity over time, which was negligible for both ES and NS.

The reactivity of settled dust, chosen to mimic dust settled on workshop floors and surfaces, of four ES dusts and one NS dust was evaluated over a 21-day timeframe. None of the ES dusts exhibited a significant change in reactivity over the 21-day timeframe. NS3 did exhibit a significant change (*p* < 0.05) in reactivity, dropping 28% over the 21-day timeframe; however, its initial reactivity was significantly lower than all ES dusts, and the decline in reactivity was relatively small in absolute terms (117 ± 12 µg/10^5^ dG on day 0 versus 84 ± 12 µg/10^5^ dG on day 21). These results suggest that ES dusts are able to maintain their relatively high reactivity for extended time periods, exceeding typical timeframes for routine housekeeping of settled dusts. This is a significant finding and has implications for industry, as it highlights the ongoing hazard associated with settled ES dust and, importantly, the need for excellent housekeeping to avoid resuspension of dusts that have settled on surfaces within the workplace.

It, of course, does not necessarily follow that such results in an acellular assay portend a toxic effect in a biological system. Shi et al., whose assay technique we have adapted, found that fine and coarse particulates induced 8-OH deoxyguanosine in A549 bronchial cells as well as in acellular systems [27]. On the other hand, Ovrevik [13], in a review of 50 studies, did not find consistent indications of a predictive effect of oxidative potential on biological effects, although none of the studies reviewed employed the deoxyguanosine assay. Our group proposes to apply this assay in a test on bronchial epithelium in future studies.

## 5. Conclusions

This study has reported the application of a deoxyguanosine assay to the assessment of ES dust reactivity. The results indicate that respirable ES dust exhibits a high overall reactivity and subsequent potential to cause oxidative damage. In general, respirable ES dust displayed higher reactivity than dust produced from two out of three natural stones and the common building material Hebel. The findings of this work show that smaller dust particles are more reactive, with freshly generated respirable dusts from ES and NS displaying significantly higher reactivity than dust that settled on surfaces after cutting events. This reinforces the importance of dust control measures in the workplace to minimise exposure to these highly reactive dusts. Further, settled ES dusts were shown to maintain their reactivity over a 21-day period. This prolonged reactivity highlights the importance of good housekeeping in workplaces to ensure that settled dusts are not resuspended into the air where they can be inhaled.

## Figures and Tables

**Table 1:** A summary of the physicochemical properties of actively generated respirable engineered and natural stone dusts assessed in this study adapted from Ramkissoon et al. [29]. Reported as average ± standard error (*n* = 3).

Stone	ID	Mineral Content				Resin	Mean Particle Size	Mean Zeta Potential	Elemental Composition					
		Quartz	Cristobalite	Total RCS	Other				Fe	Al	Ni	Ti	Co	Cr
		%	%	%	%	%	nm	mV	%	%	%	%	%	%
Engineered	ES1	86.7	4.20	90.9	0.5	8.6	630 ± 50	−26.1 ± 0.18	0.06	1.33	0.004	0.84	0.008	0.002
	ES2	42.4	44.0	86.4	1.6	12.0	533 ± 120	−32.9 ± 0.20	0.06	1.35	0.004	1.61	0.01	<0.001
	ES4	67.8	2.6	70.4	14.0	15.6	500 ± 109	−28.2 ± 0.15	0.04	0.16	0.003	0.75	0.008	0.002
	ES5	90.2	-	90.2	-	9.8	509 ± 30	−29.4 ± 0.82	0.03	0.34	<0.001	0.43	0.009	<0.001
	ES6	20.0	65.5	85.5	1.0	13.5	417 ± 169	−25.7 ± 0.86	0.13	1.07	0.004	0.07	0.006	0.002
	ES7	86.7	-	86.7	-	13.3	416 ± 25	−30.0 ± 0.95	0.04	1.33	0.02	0.84	0.008	0.002
	ES8	90.9	-	90.9	-	9.1	715 ± 91	−30.0 ± 0.72	0.05	0.47	0.006	0.57	0.006	0.004
	ES9	87.6	-	87.6	-	12.4	578 ± 44	−30.5 ± 0.62	0.11	0.35	0.01	0.22	0.01	0.004
	ES10	87.6	-	87.6	0.3	12.1	218 ± 34	−28.0 ± 0.70	<0.01	0.14	0.004	0.47	0.008	0.002
	ES11	46.4	31.4	77.8	5.8	16.4	576 ± 10	−33.8 ± 1.10	0.03	0.29	0.004	0.37	0.006	0.002
	ES12	25.4	54.6	80.0	-	20.0	455 ± 92	−26.6 ± 0.31	0.05	0.17	0.006	0.13	0.009	0.004
	ES13 ^a^	66.0	-	66.0	24.3	9.7	634 ± 48	−28.2 ± 0.80	0.37	22.5	0.007	0.47	0.03	0.03
Natural	NS1	30.1	-	30.1	69.9	NA	503 ± 11	−28.3 ± 0.55	6.63	8.36	0.02	0.04	0.002	<0.001
	NS2	3.5	-	3.5	96.5	NA	634 ± 22	−15.2 ± 0.80	0.02	<0.01	0.01	<0.01	0.001	0.01
	NS3	11.0	-	11.0	89.0	NA	575 ± 10	−22.9 ± 0.20	0.03	0.02	<0.001	0.04	0.002	<0.001
NA	Hebel ^a^	5.9	-	5.9	94.1	NA	651 ± 30	−21.7 ± 0.97	1.13	3.53	0.005	0.20	0.004	0.016

Nb. ES3 from previous study not evaluated in this work: ^a^, supplementary data acquired for new samples that have not previously been reported.

**Table 2:** Comparison of the oxidative damage potential of actively generated respirable and settled dust from engineered (ES1-13) and natural (NS1-3) stone measured using the deoxyguanosine assay. Presented as mean ± SD (*n* = 2).

Sample Type	Stone ID	8-OH-dG (µg/10^5^ dG)	
		Respirable	Settled
Other Materials	Reference RCS	14 ± 25	NA
	Hebel	312 ± 87	120 ± 6.5
Natural Stone	NS1	3305 ± 86	723 ± 103
	NS2	162 ± 4.8	21 ± 13
	NS3	948 ± 141	137 ± 22
Engineered Stone	ES1	2920 ± 435	1563 ± 33
	ES2	3201 ± 161	944 ± 140
	ES4	3184 ± 15	1945 ± 162
	ES5	3946 ± 96	1407 ± 19
	ES6	2451 ± 92	1221 ± 71
	ES7	4757 ± 853	1540 ± 750
	ES8	3019 ± 71	1006 ± 151
	ES9	2419 ± 399	871 ± 78
	ES10	2914 ± 81	767 ± 37
	ES11	2103 ± 156	1106 ± 78
	ES12	3156 ± 15	1918 ± 162
	ES13	3808 ± 4.5	723 ± 107

## Data Availability

The data presented in this study are available in Rapid assessment of oxidative damage potential: A comparative study of engineered stone dusts using a deoxyguanosine assay.
